# High-Low Impact Exercise Program Including Pelvic Floor Muscle Exercises Improves Pelvic Floor Muscle Function in Healthy Pregnant Women – A Randomized Control Trial

**DOI:** 10.3389/fphys.2018.01867

**Published:** 2019-01-30

**Authors:** Anna Szumilewicz, Marcin Dornowski, Magdalena Piernicka, Aneta Worska, Agnieszka Kuchta, Jakub Kortas, Monika Błudnicka, Łukasz Radzimiński, Zbigniew Jastrzębski

**Affiliations:** ^1^Department of Fitness and Strength Conditioning, Gdansk University of Physical Education and Sport, Gdańsk, Poland; ^2^Department of Sport Theory and Motor Skill, Gdansk University of Physical Education and Sport, Gdańsk, Poland; ^3^Department of Clinical Chemistry, Medical University of Gdańsk, Gdańsk, Poland; ^4^Department of Recreation and Qualified Tourism, Gdansk University of Physical Education and Sport, Gdańsk, Poland; ^5^Department of Anatomy and Anthropology, Gdansk University of Physical Education and Sport, Gdańsk, Poland; ^6^Department of Physiology and Pharmacology, Gdansk University of Physical Education and Sport, Gdańsk, Poland

**Keywords:** high-low aerobics, pelvic floor muscles, pregnancy, prenatal physical activity, exercise program, sEMG, biofeedback, stress urinary incontinence

## Abstract

**Background:** Pregnancy and high-impact activity are considered as risk factors for pelvic floor dysfunctions, including urinary incontinence.

**Aim:** To investigate whether a structured exercise program, including high- and low-impact aerobics and supported by pelvic floor muscle exercises, improves the neuromuscular activity of the pelvic floor and does not reduce the quality of life in terms of urinary incontinence in healthy pregnant women.

**Methods:** This was a randomized control trial among 97 Caucasian healthy nulliparas in uncomplicated pregnancies (age 30 ± 4 years, 21 ± 5 weeks of gestation; mean ± SD). Women were assessed for pelvic floor muscle functions with surface electromyography (EMG) using vaginal probes and using the Incontinence Impact Questionnaire (IIQ). Only women able to contract pelvic floor muscles and with good quality of life based on IIQ were included for the study. Seventy women in the experimental group took part in a supervised exercise program including high-low impact aerobics and pelvic floor muscle exercises three times a week. Twenty-seven controls did not receive any exercise intervention. After 6 weeks both groups were re-tested with EMG and IIQ. Post- and pre-exercise program changes in each group were analyzed using a repeated-measures ANOVA.

**Results:** Women in the experimental group improved the neuromuscular activity of the pelvic floor in some motor tasks without any adverse outcomes of the intervention. After the exercise program we observed in the experimental group significantly higher EMG amplitude in the pelvic floor muscles during 3-s contractions (*p* = 0.014). We also noticed a beneficial trend in the increase of neuromuscular activity during 10- and 60-s contractions, but the changes were not statistically significant. The exercising women substantially improved their abilities for relaxation following 3- and 10-s contractions (*p* = 0.013 and *p* < 0.001). In controls, we reported no statistically significant improvement in either of the motor tasks. All study participants maintained good quality of life related to urinary incontinence.

**Conclusion:** Prenatal exercise programs that include high- and low-impact aerobics and are supported by pelvic floor muscle exercises should be recommended for pregnant women, especially those who are accustomed to higher exercise intensity before pregnancy. Nevertheless, these recommendations can be directed to continent women who can properly contract pelvic floor muscles.

**ISRCTN. DOI: 10.1186/ISRCTN92265528:** “Pelvic floor muscle training with surface electromyography”, retrospectively registered on the 25th of July, 2016.

## Introduction

Official guidelines on exercise in pregnancy from different countries recommend aerobics for women as a form of cardio exercise, however, some of them limit this recommendation to low-impact aerobics (Szumilewicz et al., 2015). In low-impact aerobics, dance-like movements are executed while keeping at least one foot on the floor at all times. Conversely, during high-impact aerobics, more intensive movements both feet are above the floor for a while (e.g., during jumping or running). Classes mixing high- and low-impact movements are labeled as high-low aerobics (Kennedy-Armbruster and Yoke, 2014). Not much data are available on the influence of high-impact aerobics on the pelvic floor muscle functions in pregnant women.

Pregnancy and increased loading on the pelvic floor are risk factors for pelvic floor dysfunctions (Delancey et al., 2008; Sangsawang and Sangsawang, 2013), including bladder and bowel dysfunction, pelvic organ prolapse, sexual dysfunction and pelvic pain (Bo et al., 2017). Stress urinary incontinence, occurring with physical exertion, is the most common type of urinary incontinence in pregnant women and has detrimental effects on the quality of life of approximately 54.3% of this population (Sangsawang and Sangsawang, 2013).

Given that performing jumps or runs increases intra-abdominal pressure (Harman et al., 1988) and high-impact activities enhance the risk of stress urinary incontinence in female athletes (Bø, 2004), it would seem reasonable to avoid such forms of exercise during gestation. On the other hand, according to a review by Nygaard and Shaw (2016), existing literature suggests that most physical activity does not harm the pelvic floor and that existing data are insufficient to determine how strenuous activity is related to pelvic floor disorders. Many authors have observed that supervised pelvic floor muscle training during pregnancy can prevent and treat urinary incontinence, improving their sphincteric function (Mørkved and Bø, 2014). Only 6 weeks of training can be enough in this regard (Sangsawang and Sangsawang, 2016) and pelvic floor muscle exercises can be effectively incorporated into general antenatal classes (Pelaez et al., 2014). To prevent urine leakage during exercise, Bourcier (2008) proposed to use “the knack” – a quick, strong, well-timed pelvic floor muscle contraction before and during physical stress, increasing intraabdominal pressure (like jumping or running).

Rather than limiting the participation of pregnant women in high-impact activities, it is worth lowering the risk of their potential negative effects based on the current scientific and practical knowledge on the function and training of pelvic floor muscles. An intensity level that is too low in relation to low-impact activity may not be sufficient to stimulate the cardiopulmonary system (Halvorsen et al., 2013) and may not produce the desired health effects. Using high-impact movements gives the opportunity to increase quickly the intensity of exercise. It is especially important for women with high levels of exercise capacity and accustomed to participating in high-intensity classes before pregnancy. In this study, we set out to test our assumption that in healthy pregnant women, a structured exercise program including high- and low-impact aerobics and pelvic floor muscle exercises improves the neuromuscular activity of pelvic floor and does not reduce the quality of life in terms of the urinary incontinence.

## Materials and Methods

This was a randomized control trial among 97 Caucasian healthy nulliparas in uncomplicated pregnancies (age 30 ± 4 years, 21 ± 5 weeks of gestation; mean ± SD) who volunteered for the study. The eligibility criterion was a normal single pregnancy confirmed by routine obstetric consultation. Exclusion criteria were as follows: any present or previous pelvic floor dysfunctions diagnosed by health professionals, history of miscarriages over 12 weeks gestation and/or more than two successive miscarriages in the first trimester, any contraindications to physical activity according to ACOG (The American College of Obstetricians, and Gynecologists [ACOG], 2015), allergy to any materials used during the study procedure (e.g., nickel in vaginal probes), and presence of a condition or abnormality that in the opinion of the investigator would compromise the safety of the participant or the quality of the data. We also excluded women who on the pre-intervention assessment with electromyography (EMG) were unable to contract pelvic floor muscles and did not show good quality of life based on the Incontinence Impact Questionnaire (IIQ) (Corcos et al., 2002).

**FIGURE 1:**
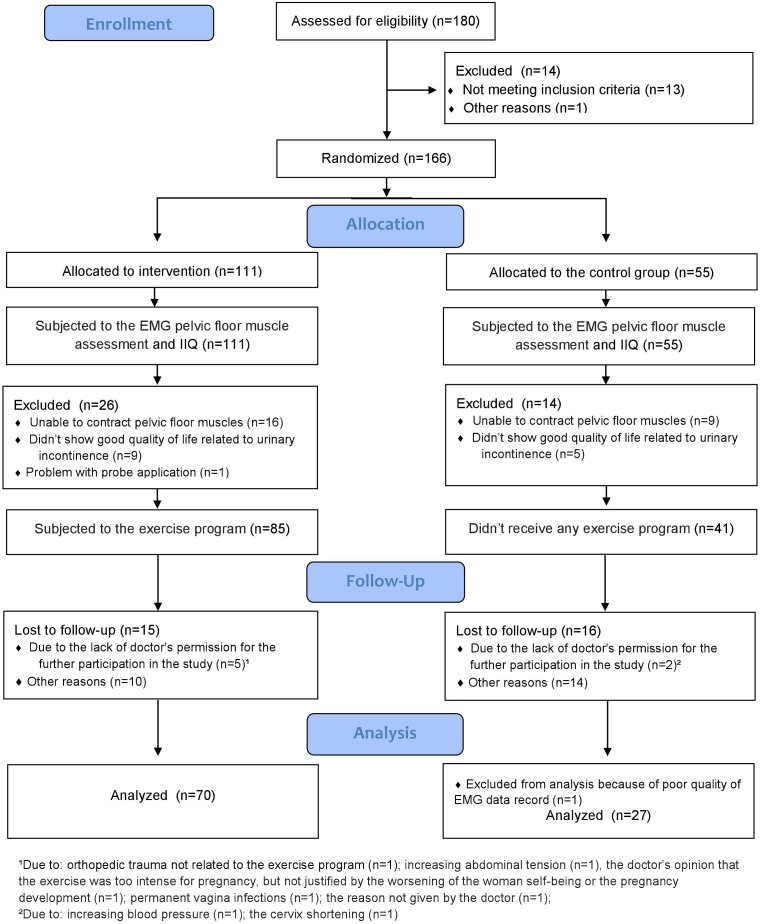
The flow of participants through the study.

The sample size was predetermined by using a power calculation with the software G^∗^power version 3.1.3. The estimated values of the mean and SD from preliminary tests with 20 exercising women allowed us to predetermine the minimal sample size for the exercising group in the performed tests, with a power of 0.8 to 29 participants. Because 160 women applied for the experiment, we decided to increase the assumed number of participants. For ethical reasons, in order to reduce the number of women left without our exercise intervention (in our opinion beneficial to women’s health), we randomized them to experimental or control groups with 2:1 ratio. For this purpose, we used STATISTICA software v. 10.0. We analyzed data from 70 and 27 women, respectively. The flow of participants through the study is presented in Figure 1.

We conducted the trial in Laboratory of Physical Effort and Genetics in Sport, at Gdansk University of Physical Education and Sport in Poland between October 2015 and May 2016. The study was performed according to the principles of the WMA Declaration of Helsinki and with the approval of the Bioethics Commission at the District Medical Chamber in Gdansk (KB - 8/14). The participants signed informed consent before testing.

### Assessment of Neuromuscular Activity of Pelvic Floor Muscles and Implementation of a Biofeedback Session

Before and after 6 weeks of intervention in both groups we performed the pelvic floor muscle assessment by surface EMG using the TeleMyo™ 2400T Direct Transmission System (DTS), NORAXON EMG and Sensors System (Scottsdale, AZ, United States). TeleMyo™ DTS complies with the requirements of the Medical Device Directive 93/42/EEC. The data were sampled at a rate of 1,000 Hz and filtered at 6–500 Hz. The sEMG data demonstrated significant test–retest reliability and clinical predictive validity for use in early detection and prophylaxis of urogynecological disorders (Glazer et al., 1999).

For the pelvic floor we used vaginal probes (Lifecare PR-02, Everyway Medical Instruments Co., Ltd., Taiwan), which were presented by Halski et al. (2014). The PR-02 probe is lightweight (23.1 g) and comfortably shaped to ensure easy hold and to prevent falling out during the contraction of pelvic floor muscles. It has a length of 76 mm and a diameter of 28 mm. It has two longitudinal recording plates (stainless steel, containing nickel) on the right and left sides. Women were asked to insert the probe into the vagina, ensuring each electrode surface pointed toward each hip. Halski et al. (2013) observed that different probe placement during functional contraction of pelvic floor muscles did not affect the obtained results in sEMG evaluation, therefore we asked the women to insert the probe as deep as they felt comfortable. We used surface disk electrodes (SKINTACT Premier W-60, LEONHARD LANG GmbH, Austria) for selected synergistic muscles: rectus abdominis, obliquus externus abdominis and gluteus maximus muscles.

We conducted the study based on SENIAM standards on EMG (Hermens et al., 1999). During the study, participants were lying in supine position with hips flexed and knees bent to approximately 90°. They performed the pelvic floor muscle contractions and relaxations: 10 s of relaxation (pelvic floor muscle EMG baseline), five 3-s maximal rapid contractions (so called quick flicks) followed by immediate relaxations, five 10-s maximal contractions followed by 10 s of relaxations and a 60-s contraction (static hold) followed by 10 s of relaxation. Other muscle groups (abdominals and gluteus) were to remain relaxed. In the test, women received the following instruction: “On command *Contract*, immediately contract your pelvic floor muscles as much as you can, keeping your abdominals, legs and buttocks relaxed, and on command *Relax*, relax your whole body.” Exercise commands were issued according to automated protocol software. After the initial assessment the experimental group received one session of verbal instructions about pelvic floor muscle contraction and relaxation while viewing the EMG (biofeedback). The control group received neither biofeedback nor verbal instruction on how to properly contract pelvic floor muscles.

The evaluation of pelvic floor muscles with surface EMG is painless and non-invasive (Glazer et al., 1999). The test protocol was developed in such a way as to ensure the greatest possible comfort with the vaginal probe application. Each participant was given the vaginal probe to be administered by herself; she went out into a separate room and, after the application, returned to the research office fully dressed. All EMG assessments were carried out in the same laboratory by a professional physiotherapist additionally trained in the urogynecological health and the use of surface EMG. Because the requirement for the trial implementation was that the experimental group attended exercise sessions three times a week, while the controls did not receive any exercise program, it was impossible to blind the study participants.

### Assessment of Urinary Incontinence Impact on the Quality of Life

Before the intervention, we used the short form of IIQ (Uebersax et al., 1995) to assess the life impact of urinary incontinence symptoms in the study participants. Based on the study by Corcos et al. (2002), we treated a score of less than 50 on the IIQ as representative of good quality of life, a score between 50 and 70 indicated moderate quality of life, and greater than 70 poor quality of life. For further analysis we included only women with good quality of life. After the 6-week intervention, we re-used the questionnaire.

### Assessment of the Exercise Capacity

At the beginning of the exercise program, all women underwent an exercise test on a cycloergometer with an electronically regulated load (Viasprint 150P). In order to establish the maximum oxygen uptake, we used a stationary respiratory gas analyzer (Oxycon Pro, Erich JAEGER GmbH, Germany). It was calibrated prior to each test according to the manufacturer’s instructions. Breath-by-breath data were averaged to provide a data point for each 15-s period. The test started with a 5-min adaptation phase when women sat on a chair. There followed a 4-min warm-up with a relative load of 0.4 W^.^kg^-1^ of body mass. After the warm up, the load increased by 0.2 W^.^kg^-1^ every minute, up to refusal. Before the experiment we instructed women to use the 0-10 Borg’s Perceived Exertion Scale (Borg, 1998). They were allowed to stop the test at any time. As women’s maximal effort, we treated the test results when they achieved the perceived exertion level of 9 or 10 and the value of respiratory exchange ratio (RER) was above 1. After the test, the participants rested for 3 min sitting on a chair. The highest oxygen uptake achieved during the maximum effort and maintained for 15 s was taken as maximal oxygen capacity (VO_2max_).

Based on the RER value we had set heart rate zones for exercise sessions. The lower heart rate limit corresponded to the RER value of 0.85. The upper heart rate limit was set at the RER value equal to 1 (Laplaud et al., 2006). Keeping the heart rate between these thresholds ensured that participants performed aerobic exercises and optimized cardiopulmonary fitness (Snow et al., 2003). Because in some women the RER values may be higher in pregnancy due to shifting metabolic energy substrates (Ajjimaporn et al., 2014), we also analyzed carefully HR curves. We have chosen aerobic exercise because, apart from numerous benefits typical for general populations, it compensates for the physiological changes in a woman’s body induced by pregnancy (Clapp, 2000).

### Prenatal Exercise Program as Experimental Intervention

The intervention group participated in the 6-week structured exercise program, designed by the principal researcher of the study according to the available guidelines (Szumilewicz et al., 2015; The American College of Obstetricians, and Gynecologists [ACOG], 2015). Group exercise sessions were held three times a week on Mondays, Wednesdays and Fridays (18 sessions) from 9.30 to 10.30 a.m. at the sport facilities of Gdansk University of Physical Education and Sport. Each session consisted of a warm-up and aerobics in the form of high- and low-impact aerobic choreography with music (25 min), strength conditioning exercises (25 min), stretching and breathing exercises and relaxation (10 min).

We taught women to consciously contract pelvic floor muscles while performing aerobics choreography and to use “the knack” especially prior to landing after a jump and any other rise in intra-abdominal pressure. To maintain proper intensity of exercise we used heart rate monitors (Polar RS400, Finland) in each session with individually adjusted heart rate zones. We trained women how to observe changes in their heart rate and to keep it within the stated ranges by increasing or decreasing the number of high impact movements (where both feet do not touch the floor for a moment). Additionally, they monitored the exercise intensity based on the “talk test” and the Rating of Perceived Exertion Scale (RPE) (The American College of Obstetricians, and Gynecologists [ACOG], 2015).

In the strengthening part, women performed nine exercises for different muscle groups in two sets of 12–16 repetitions, with a break of 30 s between sets. We instructed them to perform the repetitions until they felt unpleasant soreness of the targeted muscles. We also trained women to contract pelvic floor muscles together with other muscle groups and to consciously relax them between the strengthening exercise repetitions, after the set of exercises and also during stretching. No equipment was used during exercises and only resistance from their own bodies was applied. At the end of this part, women performed isolated pelvic floor muscle exercises (trying to keep abdominals, buttocks and tights relaxed during pelvic floor muscle contractions) according to the modified graduated strength training program by Miller (1996) (Supplementary Table S1). The program contained exercises for both contracting and relaxing pelvic floor muscles.

The sessions were conducted by a certified Pregnancy and Postnatal Exercise Specialist whose competences met the European educational standard for this profession (Santos-Rocha et al., 2016). She was informed of the aim of the study and trained in terms of monitoring and maintaining the desired intensity of exercise (inter alia by using rest breaks or implementing jumps and optional repetitions) and reminding women about the contraction and relaxation of the pelvic floor muscles at the right moments of exercise throughout the class. The principal researcher checked the quality of exercise program implementation once every 2 weeks. We used email and phone contact to ensure adherence to the program. The exercise specialist checked and registered attendance for each session.

### Primary Outcomes

To observe the post- and pre-intervention changes in neuromuscular activity of the pelvic floor muscles, we analyzed the EMG amplitude mean value for selected tasks: 10-s relaxation before the test sequence (pelvic floor muscle EMG baseline), mean of five 3-s maximal rapid contractions (quick flicks), mean of five 3-s relaxations following the quick flicks, mean of five 10-s maximal contractions, mean of five 10-s relaxations following the 10-s contractions, the 60-s contraction (static hold) and the 10-s relaxation following the static hold. For quantitative analysis we normalized the raw EMG data in μV to the individual value of maximal pelvic floor muscle contraction lasting for at least 3 s (MVC) performed by study participants during the test sequence (in further analysis expressed as % of MVC).

### Secondary Outcomes

To assess the post- and pre-intervention changes in the urinary incontinence impact on the quality of life, we analyzed the average IIQ scores.

### Statistical Analysis

Statistical analysis was performed using the Statistica software package (STATISTICA 13.1 Statsoft Poland). Variables were expressed as mean ± standard deviation (M ± SD). To compare the pre-intervention values of selected variables between groups, due to their different size, we used the Mann–Whitney test. Post- and pre-exercise program changes in each group were analyzed using a repeated-measures ANOVA. An obtained *p*-value of less than 0.05 was considered statistically significant.

To additionally support our data analysis and interpretation and to compare the experiment outcomes between the groups, we used the Hopkins’ *Pre-post parallel-groups trial spreadsheet* (Hopkins, 2006). The probability of an effect being practically worthwhile was calculated according to the magnitude-based inference approach using the following scale: 25–75%, possibly; 75–95%, likely; 95–99.5%, very likely; >99.5%, most likely (Hopkins et al., 2009). The default probabilities for declaring an effect practically beneficial were those for which the chance of benefit outweighed risk of harm (an odds ratio of benefit to harm > 66) (Hopkins, 2006). As a threshold value for the smallest important or harmful effect, we used the default of 0.2.

## Results

In Table 1, we present the characteristics of the study groups. The experimental and control groups in terms of age, week of gestation, BMI, physical fitness and exercise heart rate zones presented values that were not statistically different (Table 1). In the pre-intervention assessment we did not observe any significant differences between the experimental and control groups in any of the measured parameters of the neuromuscular activity of the pelvic floor muscles and the IIQ score. On average, women from the experimental group attended 13 ± 3 exercise sessions (from a maximum of 18), which constituted 71% ± 19 of the planned exercise program. We observed a participant loss of 18% and 39% in the experimental and control groups, respectively.

**Table 1:** Characteristics of the study participants.

Variable at baseline	All pregnant women *n* = 97 (M ±*SD*)	Experimental group *n* = 70 (M ± *SD*)	Control group *n* = 27 (M ± *SD*)	*P*-Value^∗^
Age, years	30 ± 4	30 ± 4	29 ± 3	0.08
Gestational age, weeks	21 ± 5	21 ± 5	19 ± 5	0.07
BMI, kg^.^m^-2^	23 ± 2.7	22.9 ± 2.8	23.5 ± 2.7	0.49
VO_2max,_ ml^.^kg^-1.^min^-1^	23.3 ± 3.9	23.3 ± 4.0	23.4 ± 3.8	0.87
**HR zones for exercise sessions:**				
HR lower limit (b^.^min^-1^)	127 ± 12	126 ± 11	129 ± 12	0.25
HR upper limit (b^.^min^-1^)	149 ± 12	147 ± 11	152 ± 12	0.08
	

*BMI, body mass index; VO_2 max,_ maximal oxygen capacity; HR, heart rate; ^∗^Mann–Whitney test; P ≤ 0.05 was considered statistically significant.*

The experimental and control groups in their maximal voluntary contractions of pelvic floor muscles reached the pre-intervention values of 24.85 ± 14.60 μV and 26.49 ± 19.21 μV (mean ± SD), respectively. After 6 weeks these values were lower by 13 ± 60% in the experimental group and by 12 ± 73% in controls.

**FIGURE 2:**
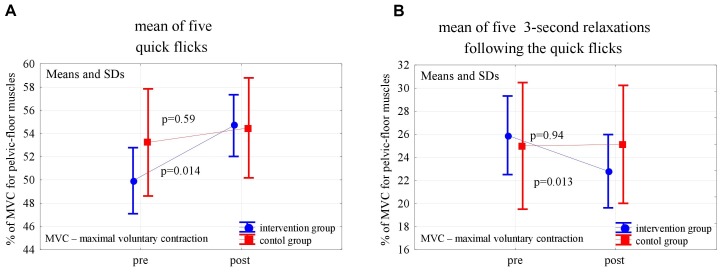
Changes in the mean EMG amplitude of pelvic-floor muscle quick flicks **(A)** and following relaxations **(B)** in the control and experimental groups after 6 weeks of high-impact exercise program.

**FIGURE 3:**
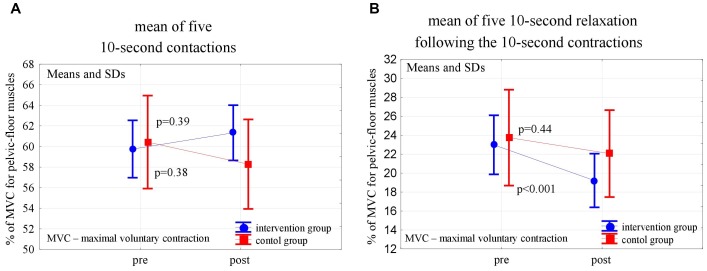
Changes in the mean EMG amplitude of the 10-s pelvic-floor muscle contractions **(A)** and following relaxations **(B)** in the control and experimental groups after 6 weeks of the high-impact exercise program.

**FIGURE 4:**
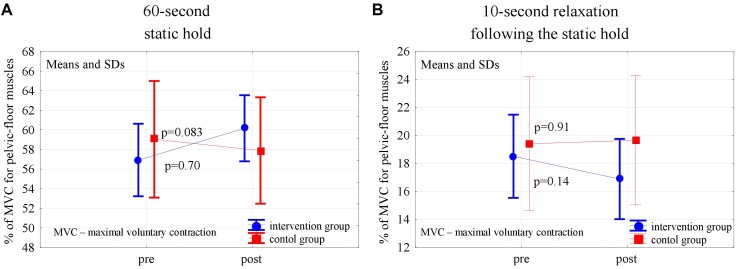
Changes in the mean EMG amplitude of the pelvic-floor muscle for the 60-s static hold **(A)** and following relaxation **(B)** in the control and experimental groups after 6 weeks of the high-impact exercise program.

In the post-intervention assessment, both in the experimental and control groups, the pelvic floor EMG activity at baseline decreased slightly, not statistically significantly, compared to the pre-intervention level (*p* = 0,24 and *p* = 0,56). After 6 weeks of the high- and low-impact exercise program, supported by complex pelvic floor muscle exercises in the intervention group, we noticed a significant increase in the EMG amplitude of quick flicks (*p* = 0.014) and a decrease in EMG amplitude in the following relaxations (*p* = 0.013). The changes were not significant in controls (Figure 2). In the 10-s contractions, the neuromuscular activity of pelvic floor muscles in both groups did not change significantly after the exercise program, but in the experimental group a significant decrease in the EMG amplitudes that followed the relaxations was noted (*p* < 0.001) (Figure 3). Similarly, despite the clear trend of increase in EMG levels for the static hold in the intervention group, the changes in both groups were not significant. Changes in the EMG amplitude of the following relaxation were also not significant in the experimental and control groups (Figure 4).

Using the probability of a practically worthwhile effect, our exercise intervention appeared to be likely beneficial for the performance of quick flicks and possibly beneficial for the 10-s contractions and following relaxations, and also for static hold. With regard to other motor tasks, the effect was trivial or unclear (Table 2).

**Table 2:** The comparison of the intervention effects between experimental and control groups in the mean EMG amplitude for pelvic floor muscle activity in selected tasks and in Incontinence Impact Questionnaire (IIQ) score.

Variables	Group	Pre-intervention level mean ± SD	Observed change mean ± SD	Probability of a practically worthwhile effect between groups^1^
**Selected tasks for pelvic floor muscles^2^:**				
10-s relaxation before the test	Control	18.71 ± 12.11	-3 ± 92%	Possibly trivial
	Experimental	17.00 ± 11.86	-8 ± 74%	
Mean of five quick flicks	Control	53.23 ± 7.86	1 ± 26%	Likely beneficial
	Experimental	50.02 ± 13.38	11 ± 37%
Mean of five 3-s relaxations following the quick flicks	Control	24.99 ± 13.01	2 ± 65%	Possibly beneficial
	Experimental	26.09 ± 14.70	-14.2 ± 68%
Mean of five 10-s maximal contractions	Control	60.43 ± 11.74	-4 ± 24%	Possibly beneficial
	Experimental	59.94 ± 11.84	2.4 ± 27%
Mean of five 10-s relaxations following the 10-s contractions	Control	23.74 ± 12.42	-7 ± 63%	Unclear
	Experimental	23.24 ± 13.46	-17 ± 55%
60-s static hold	Control	59.04 ± 15.13	-3 ± 33%	Possibly beneficial
	Experimental	56.92 ± 15.74	11.6 ± 74%
10-s relaxation following the static hold	Control	19.40 ± 13.14	-17 ± 233%	Unclear
	Experimental	18.51 ± 12.27	-11 ± 139%
**Incontinence Impact Questionnaire (IIQ)^3^:**			
Average IIQ score	Control	0.18 ± 0.92	0.12 ± 0.93%	Very likely trivial
	Experimental	2.24 ± 7.76	0.20 ± 7.58%

*Experimental group participated in supervised prenatal exercise program, including high-low impact aerobics and pelvic floor-muscle exercises three times a week (n = 70). Control group didn’t receive any treatment (n = 27). ^1^The probability of an effect being practically beneficial was calculated according to the magnitude based inference approach. The unclear outcome means that more data are needed to assess the intervention effect. ^2^Expressed as % of Maximal Voluntary Contraction (MVC). ^3^Expressed in raw score.*

In the initial assessment, all women in both groups presented good quality of life based on the IIQ (average score < 50 for a maximum of 100), as this was our criterion to allocate women to this trial. Nine women from the experimental group reported some symptoms of urinary incontinence (6%; 18 ± 8 IIQ score) and one from the controls (3%; 5 IIQ score). After 6 weeks the average IIQ scores did not change significantly (*p* = 0.81 and *p* = 0.59, respectively). In the post-intervention assessment, nine women from experimental group still reported some symptoms of urinary incontinence (6%; 16 ± 13 IIQ score), and in controls the number increased to two symptomatic women (6%; 5 ± 0 IIQ score). All women in both groups maintain good quality of life.

We have noted no adverse effects on the participant’s health or well-being or on the course of pregnancy related to the implementation of the exercise program.

## Discussion

The main finding of this study is that women participating in a structured exercise program including high- and low-impact aerobics and pelvic floor muscle exercises improved the neuromuscular activity of the pelvic floor in some motor tasks. Their EMG levels significantly increased in quick flicks. We also noticed a beneficial trend in the increase of neuromuscular activity during 10-s contractions and 60-s static holds, but the changes were not statistically significant. Moreover, after 6 weeks of exercising the experimental group substantially improved their abilities for relaxation following quick flicks and 10-s contractions. In contrast to these results, in the control group we reported no statistically significant improvement in either of the motor tasks. Although our study did not confirm a significant positive impact from our intervention on all the analyzed motor skills, it is certainly evidence that performing high-impact exercise by healthy pregnant women does not negatively affect their neuromuscular activity of pelvic floor. Therefore, we can attempt to refute anecdotal opinion that such physical activity should always be avoided during gestation. On the other hand, even small improvements in pelvic floor functions in our study participants should be treated as a success. Despite the positive factor of exercise, at the same time negative factors stemming from a growing uterus and pregnancy hormones influence the condition of the pelvic floor muscles. The effectiveness of our intervention may result inter alia from the supervision of an exercise specialist during each exercise session. Based on the systematic review, Mørkved and Bø (2014) found that pelvic floor muscle training is effective when the training is supervised. Another important factor is the pre-intervention assessment of pelvic floor muscle contraction. Pelaez et al. (2014) stated that in their experimental trial among pregnant women, they could have expected a stronger effect of exercise program on the decrease in urinary incontinence if there had been individual assessment of the ability to contract the pelvic floor muscles. In previous studies the proportion of women who were unable to correctly activate the pelvic floor muscles on initial testing varied from 14 to 53% (Henderson et al., 2013; Kandadai et al., 2015; Vermandel et al., 2015). Only two-thirds were confident that they were doing pelvic floor muscle exercises correctly (Chiarelli et al., 2003), while at least one in five may have had a mistaken belief in their confidence (Vermandel et al., 2015). Because of the inability to contract the pelvic floor on initial EMG assessment, we excluded 25 women (15%) from this study: 16 women (14%) from the experimental group and 9 (17%) from the control group. We wanted to increase the probability that our participants would consciously use the pelvic floor muscles to prevent urine leakage during high-low activities. We also followed the recommendation of some authors to use biofeedback in pelvic floor muscle training (Arnouk et al., 2017). The experimental group confirmed the correctness of their pelvic floor muscle contractions and relaxations with an EMG biofeedback session before the exercise program implementation.

A positive finding is that participation in the high- and low-impact exercise program did not negatively affect incontinence in pregnant women. We even observed a trend toward lowering the IIQ score after the exercises in the experimental group, but this result was not substantial. The studies by other authors confirm that low-impact aerobic exercise, supplemented with pelvic floor muscle exercises (Stafne et al., 2012; Pelaez et al., 2014) or isolated pelvic floor muscle training under the supervision of a physiotherapist (Sangsawang and Sangsawang, 2016), is effective in the treatment of urinary incontinence during pregnancy. Our outcomes indicate that for asymptomatic pregnant women, high-impact exercise supported by pelvic floor muscle training is safe in respect of urinary incontinence.

To avoid the potential negative influence of high-impact activity on the pelvic floor, the crucial element of our intervention was incorporating the pelvic floor muscle exercises from the very beginning and throughout the entire exercise session. The exercise specialist taught women to use “the knack” in the cardio part, during marching and aerobics movements, and especially in jumps or runs. Miller et al. (2008) have confirmed that the knack maneuver has immediate effect in preempting cough-related stress incontinence. Our study participants used it for the prevention of urine leakage during the exercises that noticeably increased the intra-abdominal pressure and load of the pelvic floor. Taking into account their very low post-intervention IIQ scores, this strategy seems to be effective for maintaining continence in active women. But this observation requires further investigation, e.g., through the pad test directly after the high- and low-aerobics session.

It is important to enable pregnant women to participate in exercise programs more strenuous than low-impact activities, which might have a positive influence on their metabolism, as well as to maintain or improve their pelvic floor muscle functions. In previous work, we have shown that in women participating in the high-low impact prenatal exercise program, the myokine irisin may improve levels of glucose homeostasis markers and compensate for metabolic changes induced by pregnancy (Szumilewicz et al., 2017). A key assumption for these outcomes was to maintain exercise intensity below an anaerobic threshold. In this study, we also individually determined the participant’s exercise heart rate zones and monitored exercise intensity using objective instruments, which was one of the study advantages. The SDs for the mean of the lower and upper HR limits and VO_2max_ (Table 1) showed that the participants’ levels of physical fitness were diverse. Thus, to maintain the same relative intensity in all women, their training stimuli should have been differentiated. It seems to be a common situation for most group activities. A skilled instructor is able to teach low- or high-impact options of movements in one choreography so that even in group classes, it is possible to adjust the intensity of exercise to the individual needs of the participants (Kennedy-Armbruster and Yoke, 2014). Therefore, supervised high- and low-impact aerobics should be recommended for group exercise classes, including those with pregnant women. In the promotion of this type of physical activity, it is worth considering also their other benefits. According to Owe et al. (2016) pre-pregnancy regular participation in high-impact exercises such as running, jogging, orienteering, ballgames, netball games and high-impact aerobics were associated with a 14% lower risk of developing pelvic girdle pain during pregnancy (aRR 0.86, 95% CI 0.77 to 0.96). It can be assumed that the continuation of these activities during gestation will be equally positive, although this requires testing in experimental studies.

In the second part of the exercise sessions, women were to activate pelvic floor muscles while strengthening other muscle groups. In the last part they performed isolated pelvic floor muscle contractions (consciously relaxing synergists) and relaxations. After such a complex intervention, we observed substantially higher EMG activity during quick flicks and a beneficial trend in the increase of neuromuscular activity during 10-s contractions and 60-s static hold, potentially related to the improvement of the pelvic floor muscle power, strength and endurance (Newman, 2014). It is probable that, owing to the versatility of our exercise program, the participants could effectively use the continence mechanism in the functional activities, diverse in terms of duration and intensity. An important task for the women was performing also relaxing exercises which are recommended inter alia to improve voiding and relieve pelvic pain caused by muscle spasms (Newman, 2014). We observed that women in the experimental group significantly decreased their neuromuscular activity during relaxations. For pregnant women, the ability to volitionally relax pelvic floor muscles seems to be particularly important to assist normal birth. This issue is worth further research.

After a 6-week intervention our participants in both groups presented similar lower mean EMG amplitudes in maximal voluntary contraction of pelvic floor muscles. These drops may have resulted from the development of pregnancy and natural maternal adaptations in preparation for parturition in vaginal support (Oliphant et al., 2014). Resende et al. (2012) found that the MVC in a pelvic floor muscle EMG exam was 30 μV for pregnant women compared to 90.7 μV in nulliparous women. For our analyses it was important that the data on neuromuscular activity of the pelvic floor, particularly the data regarding motor tasks before and after the intervention, were normalized to the MVC value. Thus, the presented outcomes reflect the degree of the use of the pelvic floor muscles in relation to the individual maximum possibilities and test conditions on a given day.

The strengths of the presented study are the randomized control trial design, the exercise program design according to ACOG recommendations and its implementation by a pre- and postnatal exercise specialist who met the requirements of European educational standards for this profession. The weak point is the control group, which consisted of women who volunteered for the prenatal exercise program. Despite the fact that as a result of randomization women from the control group were not subjected to our exercise program, it is still possible that in response to their interest they undertook physical activity on their own, including pelvic floor muscle exercises. Considering that physical inactivity in pregnancy is a risky behavior (Halvorsen et al., 2013), for ethical reasons we did not ask the control group to refrain from the exercises during the experiment. It would be interesting to compare the pelvic floor muscle function of pregnant women who underwent the high- and low-impact exercise program with a group more representative for the total population of women. It can be assumed that the differences between such groups would be greater than the results presented in this study. Another limitation is that it is impossible to assess which part of the exercise intervention influenced the pelvic floor muscle functions most; was it the aerobic, strengthening or relaxation parts, or just the pelvic floor muscle exercises? However, in our opinion such a complex program may lead to many other health benefits for the mother and the fetus, although this was not analyzed in this study. Therefore, due to ethical considerations, we decided not to subject women to selected parts of the program, e.g., only to pelvic floor muscle training.

To increase the credibility of our research, it would be valuable to assess the pelvic floor muscle functions in an upright position and to use objective methods to assess the incidence of urinary incontinence and its severity. Although the IIQ is a validated tool, recommended for clinical and research applications (Uebersax et al., 1995), it is possible that not all symptoms of urinary incontinence were properly recorded by the study participants. Another limitation of our work is that we do not know the proportion of low- and high-impact movements performed by women during the exercise sessions. The participants had the task of maintaining the determined aerobic intensity within the individual exercise HR zones. They decided how often and for how long they would perform low or high options of movements, following the suggestions of the exercise specialist. The purpose of the exercise program was not to achieve a specific pelvic floor muscle load through high-impact movements, but physical effort with health-promoting intensity. Setting such a goal is typical for the practice of instructors and trainers of prenatal exercises. It should be emphasized that in the study group we did not observe any women who would not perform any jumps or runs during aerobics choreography.

## Conclusion

Taking into account the positive impact of the prenatal exercise program including high-and low-impact aerobics and pelvic floor muscle exercises on the pelvic floor muscle functions, it should be recommended for pregnant women. This is especially true for those who were accustomed to a higher intensity of physical exertion before pregnancy. Nevertheless, these recommendations can be directed to women with a good quality of life in terms of the urinary incontinence and who can properly contract the pelvic floor muscles.

## Data Availability

The full trial protocol and raw data supporting the conclusions of this manuscript will be made available by the authors, without undue reservation, to any qualified researcher.

## Author Contributions

AS: trial coordinator, trial design, prenatal exercise program design and supervision of its implementation, data collecting, statistical analyses, manuscript preparation, and funding. MD: supervision of EMG assessment and contributed to the EMG test protocol design. MP: EMG assessment and data collecting. AW: prenatal exercise program implementation. AK and JK: statistical analyses. MB: EMG data collecting. ŁR: exercise capacity assessment. ZJ: the protocol design for the exercise capacity assessment and supervision of exercise capacity assessment. All authors contributed and approved the final version of the manuscript.

## Conflict of Interest Statement

The authors declare that the research was conducted in the absence of any commercial or financial relationships that could be construed as a potential conflict of interest.
