# Supplementary material for: High-Low Impact Exercise Program Including Pelvic Floor Muscle Exercises Improves Pelvic Floor Muscle Function in Healthy Pregnant Women – A Randomized Control Trial
Source: Front Physiol. 2019 Jan 30;9:1867. doi: 10.3389/fphys.2018.01867 (PMC6363702; doi:10.3389/fphys.2018.01867)
Supplement: Supplementary file 1 [file Table_1.PDF]

Pelvic floor muscle exercises for study participants

Week number	Exercise	Repetitions	Frequency and duration
Week 1	**„Quick flicks"**<br>**Goal:** learning to activate pelvic floor muscles through short contractions.<br>**Performance:** Avoid contracting the abdominal, gluteal and thigh muscles or spine movement	10 short contractions in a series.<br>5 series with breaks of 30 seconds between the series.	~5 minutes at the end of general exercise session/3 times a week with the supervision of an exercise specialist
Week 2	**„Stacking"**<br>**Goal:** fuller activation of the pelvic floor muscles through short, increasingly stronger contractions.<br>**Performance:** Each repetition of the exercise contains 3 increasingly stronger contractions (flicks) and slow reduction in tension up to complete relaxation. Count: '1, 2 3 – relax', in each repetition of the exercise.	10 repetitions in a series.<br>5 series with breaks of 30 seconds between the series.	~5 minutes at the end of general exercise session/3 times a week with the supervision of an exercise specialist
Week 3	**„Endurance"**<br>**Goal:** maintenance of sustained holds of moderate intensity.<br>**Performance:** Each repetition consists of 3 increasingly stronger flicks, maintaining sustained hold, gradually extending the duration (from 3 to 10 seconds), and then slowly reducing the tension up to full relaxation.<br>The contraction of the pelvic floor should be directed upwards and inwards. During the sustained holds, the pelvic-floor muscles should be activated as strongly as possible, without contracting other groups of muscles.	10 repetitions in a series.<br>10 sec. breaks between repetitions, contraction at the highest level to be kept for 3 – 10 seconds (gradually extending the duration together with the increase in the muscle power).<br>3 series with breaks of 30 seconds between the series.	~10 minutes at the end of general exercise session/3 times a week with the supervision of an exercise specialist
Week 4	**„High-Intensity"**<br>**Goal:** maximum hold of high intensity in order to increase the mass and power of the muscles.<br>**Performance:** Each exercise repetition consists of 3 increasingly stronger flicks, maintaining maximum hold until the feeling of tiredness, execution of 5 pulsating	5 repetitions in a series, 10 seconds breaks between repetitions, maintenance of maximal hold (above 10 seconds) extending it by short pulsating	~10 minutes at the end of general exercise session/3 times a week with the supervision of an exercise specialist

	flicks before relaxing and then slow reduction of tension up to full relaxation.	contractions before relaxation.<br>3 series, with 30s breaks between the series.	
Week 5	**„Complex activation"**<br>**Goal:** improving speed, strength and endurance of the pelvic-floor muscles. Increasing body awareness and differentiating moment of tension and relaxation of muscles.<br>**Performance:** One sequence of "complex activation" contains: 5 quick and maximal contractions with immediate relaxation (5 second pause between contractions), 5 maximal contractions (10 seconds contraction, 10 seconds pause) sustained hold lasting 60 seconds with immediate relaxation after the long contraction.	3 sequences of contractions and relaxation, 30s pauses between series.	~10 minutes at the end of general exercise session/3 times a week with the supervision of an exercise specialist
Week 6	**„Maintenance"**<br>**Goal:** Improving the ability of activating the pelvic-floor muscles in daily activities. Work on the awareness of contracting the pelvic-floor muscles before sneezing, coughing, lifting objects. Maintaining optimal health of the pelvic-floor muscles by performing regular tasks developed for week 4.	5 repetitions in a series, 10 seconds breaks between repetitions, maintenance of maximal hold (above 10 seconds) extending it by short pulsating contractions before relaxation.<br>2 or more series, with 30 seconds breaks between the series.<br>Contracting pelvic floor muscles each time before sneezing, coughing and lifting objects.	~5-10 minutes at the end of general exercise session/3 times a week with the supervision of an exercise specialist<br>and contracting pelvic floor muscles each time before sneezing, coughing and lifting objects throughout the day

Above program is based on:

Miller, J. M. (2012). Graduated strength training: A pelvic muscle exercise program.Retrieved from http://www.med.umich.edu/1libr/HealthyHealing/GraduatedStrengthTraining.pdf
